# Improved selection of participants in genetic longevity studies: family scores revisited

**DOI:** 10.1186/s12874-020-01193-7

**Published:** 2021-01-06

**Authors:** Mar Rodríguez-Girondo, Niels van den Berg, Michel H. Hof, Marian Beekman, Eline Slagboom

**Affiliations:** 1 Department of Biomedical Data Sciences, section of Medical Statistics, Leiden University Medical Center, Albinusdreef 2, 2333 ZA Leiden, the Netherlands; 2 Department of Biomedical Data Sciences, Section of Molecular Epidemiology, Leiden University Medical Center, Albinusdreef 2, 2333 ZA Leiden, the Netherlands; 3 Department of Clinical Epidemiology, Biostatistics, and Bioinformatics, Amsterdam UMC, University of Amsterdam, Meibergdreef 9, 1105 AZ Amsterdam, the Netherlands

**Keywords:** Longevity, Mixed effects modelling, Family history score, Family size

## Abstract

**Background:**

Although human longevity tends to cluster within families, genetic studies on longevity have had limited success in identifying longevity loci. One of the main causes of this limited success is the selection of participants. Studies generally include sporadically long-lived individuals, i.e. individuals with the longevity phenotype but without a genetic predisposition for longevity. The inclusion of these individuals causes phenotype heterogeneity which results in power reduction and bias. A way to avoid sporadically long-lived individuals and reduce sample heterogeneity is to include family history of longevity as selection criterion using a longevity family score. A main challenge when developing family scores are the large differences in family size, because of real differences in sibship sizes or because of missing data.

**Methods:**

We discussed the statistical properties of two existing longevity family scores: the Family Longevity Selection Score (*FLoSS*) and the Longevity Relatives Count (*LRC*) score and we evaluated their performance dealing with differential family size. We proposed a new longevity family score, the *mLRC* score, an extension of the *LRC* based on random effects modeling, which is robust for family size and missing values. The performance of the new *mLRC* as selection tool was evaluated in an intensive simulation study and illustrated in a large real dataset, the Historical Sample of the Netherlands (HSN).

**Results:**

Empirical scores such as the *FLOSS* and *LRC* cannot properly deal with differential family size and missing data. Our simulation study showed that *mLRC* is not affected by family size and provides more accurate selections of long-lived families. The analysis of 1105 sibships of the Historical Sample of the Netherlands showed that the selection of long-lived individuals based on the *mLRC* score predicts excess survival in the validation set better than the selection based on the LRC score .

**Conclusions:**

Model-based score systems such as the *mLRC* score help to reduce heterogeneity in the selection of long-lived families. The power of future studies into the genetics of longevity can likely be improved and their bias reduced, by selecting long-lived cases using the *m*LRC.

## Background

There is strong evidence that longevity, defined as survival to extreme ages, clusters within families and is transmitted across generations [1–7]. Recent research [5] on two large population-based multi-generational family studies indicates that longevity is transmitted as a quantitative genetic trait. Moreover, associations between environmental factors and familial clustering have been rarely found using historical pedigree data [5, 8–10]. Although these findings suggest that human longevity has a genetic component, genetic studies on longevity have had limited success in identifying longevity loci [11–17]. One of the main causes for this limited success could be the large heterogeneity in criteria for participant selection in longevity studies [5, 18, 19]. Since the study participants must be alive to extract blood or other biomaterials their longevity phenotype is, by definition, unknown. An additional complication of longevity studies is the ongoing increase in life expectancy due to non-genetic factors [20], such as improvements in nutrition, life style and health care. If only individual age is considered as selection criterion, these non-genetic factors increase the risk of including sporadically long-lived individuals i.e. individuals with the longevity phenotype but who do not have an underlying genetic predisposition for longevity.

To obtain a sample with less phenotype heterogeneity, the family history of longevity can be used as a participant selection criterion [5, 18]. Although this approach does not avoid that sample selection is influenced by family-shared non-genetic factors potentially involved in longevity, it is likely that it increases the power in case-control studies to detect novel genetic loci [21, 22]. A natural way to incorporate family history in the study design is to develop a longevity family score to identify families with the heritable longevity trait and to subsequently select alive members of these families for (genetic) longevity studies. A number of longevity family scores have been previously proposed [4, 18, 23–25], using different definitions of individual longevity and different ways of summarizing longevity within families. The implications of these choices are not well understood, namely how the interplay among individual longevity definition, family-specific summary measures and family size affects the sample selection process based on longevity family scores. The first challenge when developing longevity family scores is defining individual longevity. It is unclear how extreme the age at death must be to label an individual as long-lived and which scale is most beneficial so that scores reflect differences in extreme survival and not just in overall lifespan. The second challenge when developing longevity family scores are the large differences in family size. These differences imply that the available information per family differs. For a family with 12 members, for instance, more information is available than for a family with 2 members only. Importantly, we typically do not know whether these differences are real differences in sibship sizes or the result of missing data caused by limitations of the data collection. If not properly addressed, differences in family size can lead to biased rankings of long-lived families. This can lead to an increased heterogeneity among selected participants in longevity studies and hence reduce power of analyses. Instead of studying the genetics of longevity, biased selections can potentially lead to the combined analysis of the genetics of longevity, fertility and other factors affecting family size, such as, for example, socio economic status. Up till now, this important challenge has not received enough attention and how to address this problem still remains open.

In this paper, we investigate to what extent existing longevity family scores such as the Family Longevity Selection Score (*FLoSS*) [23] and the Longevity Relatives Count (*LRC*) score [18], are affected by differential family size. Subsequently, we propose an alternative method based on mixed effects regression modelling to deal with differences in family size when building a longevity family score.

The main novelty of our new approach is to consider the family size as a source of uncertainty when estimating the level of longevity of a family. Hence, we propose to select families accounting for such estimated uncertainty. This new approach will contribute to more robust scores and selection rules in longevity studies.

## Methods

### Existing longevity family scores and family size

Several longevity or excess survival family scores have been previously proposed [4, 18, 23–25]. Often, to measure individual survival exceptionality, age at death is transformed to the corresponding survival percentile [18] or related measure such as the cumulative hazard [4, 23, 25] using life table data of a reference population, typically matching for sex and birth cohort. An alternative approach based on defining individual survival exceptionality as the difference between individual’s age at death and the sample-based expected age at death correcting for a number of confounders has been also proposed [24].

We focus on two of the previous proposals, representative of two different ways of summarizing individual survival exceptionality within families: the Family Longevity Selection Score (*FLoSS*) [23] and the Longevity Relatives Count (*LRC*) score [18]. The *FLoSS* relies on a sum to summarize survival exceptional within families, while the *LRC* score is representative of the rest of previously proposed longevity scores which all rely on an empirical expectation as summary, i.e., the mean [4, 24, 25] or a proportion [18] depending on the nature of the individual measure of survival exceptionality. These two type of summary measures (sum versus empirical expectation) have different implications with regard to the influence of family size in the resulting scoring system.

### The *FLoSS* favors large families

The Family Longevity Selection Score (*FLoSS*) [23] was constructed using siblings included in the Long Life Family Study. The *FLoSS* is a modification of the *SE*_*f*_ score which adds a bonus for the presence of living family members. Since the main properties of *SE*_*f*_ transfer to *FLoSS*, for the sake of simplicity we focus on the properties of the *SE*_*f*_, defined, for each family *i*, as follows:

$$ {SE}_{fi}=\sum \limits_{j=1}^{N_i}{SE}_{ij}=\sum \limits_{j=1}^{N_i}\left(-\mathit{\log}\left(S\left({t}_{ij}|{bc}_{ij},{sex}_{ij}\right)\right)-1\right)=\sum \limits_{j=1}^{N_i}\left(\varLambda \left({t}_{ij}|{bc}_{ij},{sex}_{ij}\right)-1\right), $$

where *t*_*ij*_ is the age at death of family member *j* of family *i*, with *j = 1,…,N*_*i*_ members, *S*(*t*_*ij*_| *bc*_*ij*_, *sex*_*ij*_) is the survival probability at age *t*_*ij*_ given sex and birth cohort in the reference population and *Λ*(*t*_*ij*_| *bc*_*ij*_, *sex*_*ij*_) is the corresponding cumulative hazard. *SE*_*ij*_ varies between − 1 (if *S*(*t*_*ij*_| *bc*_*ij*_, *sex*_*ij*_) = 1) and ∞ (if *S*(*t*_*ij*_| *bc*_*ij*_, *sex*_*ij*_) = 0). The maximum value of *SE*_*ij*_ is determined by the maximum age recorded in the used life table. If for example, this maximum age at death is 99, like in the Dutch life tables [26], and the minimum survival in the population is *S*(99| *bc*_*ij*_, *sex*_*ij*_) = 0.01, this provides a maximum *SE*_*ij*_ = 4*.*6. The reference value, corresponding to a value *SE*_*ij*_ = 0 corresponds to *S*(*t*_*ij*_| *bc*_*ij*_, *sex*_*ij*_) = 0.37. This means that family members with age at death beyond the top 37% survivors count positively in the score and those with younger ages at death count negatively. For example, using the Dutch life tables, this cut-off would correspond, for those born around 1900 with an age of death of around 73 years for men and of around 80 for women. This thresholds are not in line with recent evidence indicating that higher ages at death need to be considered to capture the heritable longevity trait [5, 18]. This problem can be solved by conditioning survival to being alive at certain age. For example, a conditioning age of 40 years has previously been proposed [23], which increases the age cut-off associated to *SE*_*ij*_ = 0. For example, using Dutch lifetables this would correspond to a cut-off of around 84 years for women and 78 year for men for individuals born around 1900. These ages correspond with percentiles survivals at birth of around 0.28 (oldest 28% survivors of their birth cohort) which are likely not extreme enough to capture the heritable longevity trait. This drawback is somehow compensated by the strongly skewed distribution of *SE*_*ij*_, meaning that the impact of increasing, for example, from 95 to 96 years is greater than the increase from 70 to 71.

**Fig. 1:**
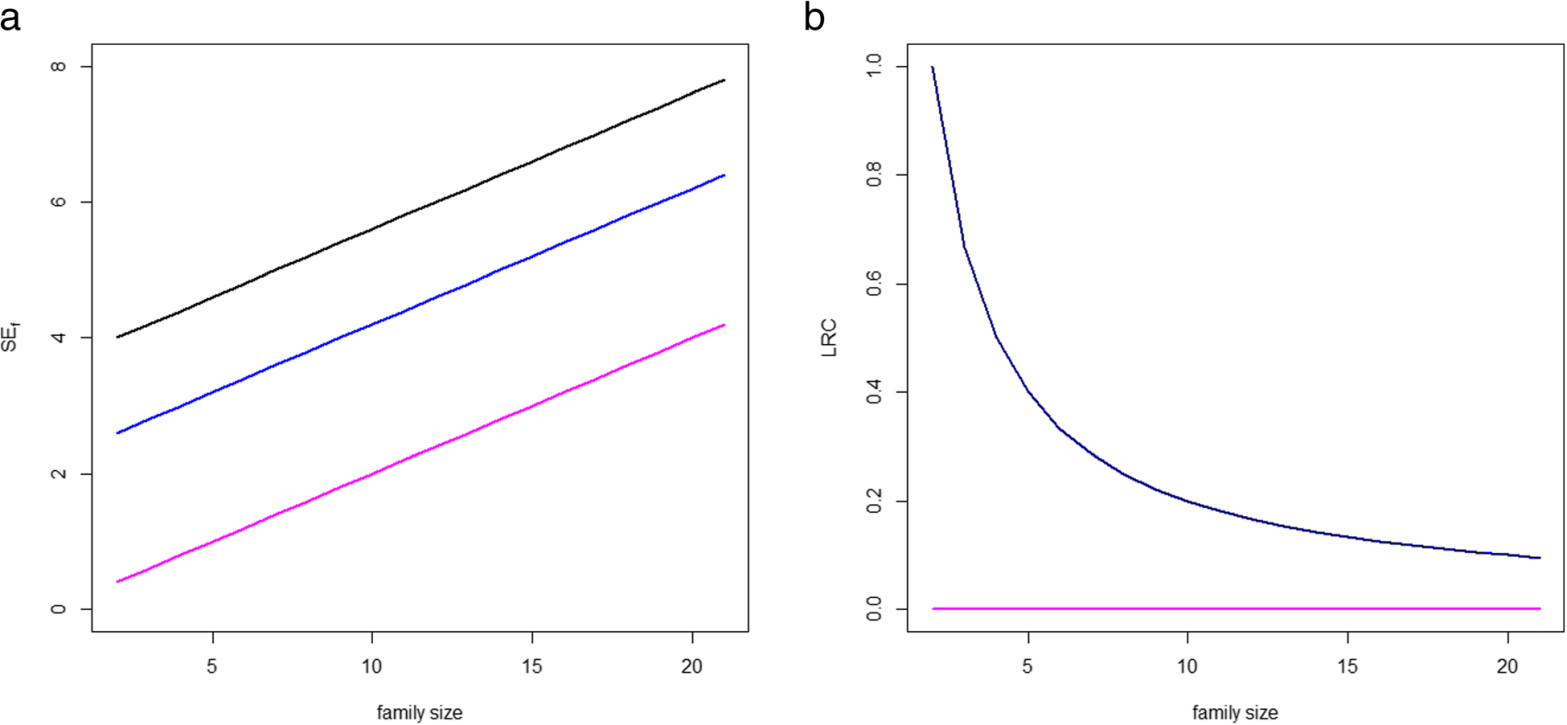
Example of three hypothetical populations with 20 sibships with sizes N_i_ = 2,3,...,21. In each population families are ranked according to *SE*_*f*_ (left panel) and *LRC* (right panel). The black lines represents a population in which all families have two siblings belonging to the top 5% survivors (long-lived) of their sex-specific birth cohort and the rest of siblings belonging to the top 30% survivors (not long-lived). The blue lines represent a population in which all families have two siblings belonging to the top 10% survivors (long-lived) of their sex-specific birth cohort and the rest of siblings belonging to the top 30% survivors. The pink lines represent a population composed of families with all family members not log-lived, belonging to the top 30% survivors. The left panel shows the value of *SE*_*f*_ with increasing number of non-lived family members. The right panel shows the value of *LRC* with increasing number of non-lived family members. Because of the definition of *LRC*, black and blue lines coincide in the right panel

An additional problem of the *SE*_*f*_ score is that it uses the sum over the available family members to summarize the level of survival exceptionality within the family. This implies that large families are systematically overweighted when using *SE*_*f*_. This phenomenon is illustrated in Fig. 1. Three example populations with twenty sibships each and different level of enrichment for longevity are considered. In the three examples, we consider sibships of increasing size, *N*_*i*_ *= i + 1*, *i = 1,2,...,20*. In the first example population, all sibships have two siblings belonging to the top 5% survivors of their sex-specific birth cohort and the rest of siblings belonging to the top 30% survivors, so these family members are clearly not long-lived. In the second, all sibships have two siblings belonging to the top 10% survivors of their sex-specific birth cohort and the rest of siblings belonging to the top 30% survivors. In the third example population all siblings belong to the top 30% survivors, representing a population with no long-lived individuals. The left panel of Fig. 1 illustrates the performance of the score *SE*_*f*_ in these three examples. Overall, increasing the sibship size leads to larger values of *SE*_*f*_*.* Moreover, larger families with lower proportions of long-lived members can present a larger value of *SE*_*f*_ than small families with a larger proportion of long-lived members. For example, a family with two members belonging to the top 10 survivors and 8 extra not long-lived siblings has a larger *SE*_*f*_ than a family with two members in the top 10 survivors and 5 extra not long-lived siblings (black line). It can also happen that a large family where two siblings are top 10% survivors and the rest not long-lived present a larger *SE*_*f*_ than a smaller family where two siblings are top 5% and the rest are not long-lived. The increasing pink line corresponding to the third scenario illustrates that large families with no long-lived family members can present large values of *SE*_*f*_, with *SE*_*f*_ arbitrarily increasing in parallel to family size.

In summary, using *SE*_*f*_ and *FLoSS* in the selection of long-lived families may lead to an overrepresentation of large families and hence undesirable heterogeneity in the selected sample of families. Importantly, the size of the families governs the range of variation of the family score implying that *SE*_*f*_ and *FLoSS* are not comparable when calculated in populations with different underlying family size patterns. Since this is an highly undesired feature, we will not further focus on the *SE*_*f*_ score (and *FLoSS*) in the rest of the paper*.*

### The *LRC* score favors small families

To mitigate the previously explained bias towards large families, a solution is to use a different summary measure at the family level, like the average [4, 25].

In this line, and based on the results of a recent study which shows that longevity is heritable beyond the 10% survivors of their birth cohort [5], the Longevity Relatives Count (*LRC*) score has been proposed [18]. The original definition of the *LRC* score allows for the inclusion of family members with different degree of relatedness. Here, we focus on its simplest form considering only siblings in its construction:

1 $$ {LRC}_i=\frac{\sum \limits_{j=1}^{N_i}I\left({P}_{ij}\ge 0.9\right)}{N_i} $$

where *P*_*ij*_ is the sex and birth cohort specific percentile survival of individual *j* of family *i*, i.e., *P*_*ij*_ = 1 − *S*(*t*_*ij*_| *bc*_*ij*_, *sex*_*ij*_). *I*(*P*_*ij*_ ≥ 0.9) is a variable indicator taking value 1 if individual *j* belongs to the top 10 survivor of his/her sex-specific birth cohort and 0 otherwise. As a result, *LRC*_*i*_ is the proportion of members of family *i* belonging to the group of top 10 survivors, defined as long-lived. The *LRC* is bounded between 0 and 1, providing a clear interpretation and comparability across populations. A drawback is that it is based on a binary definition of longevity, ignoring differences in longevity beyond the top 10% of survivors.

The *LRC* score is based on calculating a proportion, and as a consequence, the resulting ranking based on this score indirectly favors small families. For small families, it is more easy to have 100% of its family members in the top 10% survivors for than large families. Hence, in small families it can be questioned whether a large *LRC* truly captures the heritable longevity trait.

The problem of this approach is of different nature than the case of the *SE*_*f*_ score. While adding not long-lived family members implies an increase in *SE*_*f*_*,* this is not the case for *LRC* (Fig. 1, right panel). Instead of a systematic bias, we now face a problem of different uncertainty levels depending of the size of the family which cannot be properly captured by an empirical proportion. Consider the following example for illustration. Two families, both with half of the siblings long-lived, but in the first case the sibship size was 2 and on the second case the sibship size was 10. It is clear that there is more information in the second case and hence the ranking should also take this into account. However, using empirical proportions small families are benefitted.

### Accounting for uncertainty in longevity family scores

To deal with the heterogeneity in information between families caused by their size, we propose to use mixed effects regression modelling in the estimation of family scores. In particular, we focus on the *LRC*, and extend its concept by introducing family specific random effects.

Let *Y*_*ij*_ = *I*(*P*_*ij*_ *≥ c*) be a binary random variable that indicates if *P*_*ij*_ is equal of larger than c, where *P*_*ij*_ is the percentile survival of individual *j* of family *i*, and *c* is a pre-specified threshold of longevity. For example, *c = 0.90*. Let *u*_*i*_ be a random effect shared by the members of the same family that reflects the unobserved factors contributing to longevity.

Assuming that *Y*_*ij*_ follows a Bernoulli distribution, the family specific probability to reach *c* is given by the following logistic regression model with random intercept:

2 $$ {p}_i=P\left({Y}_{ij}=1|{u}_i\right)=\frac{e^{\beta_0+{u}_i}}{1+{e}^{\beta_0+{u}_i}} $$

We assume that *u*_*i*_ follows a normal distribution with mean zero and variance *σ*^2^. Then, the parameters *β*_0_ and *σ*^2^ can be estimated maximizing the resulting likelihood function $\prod \limits_{i=1}^N{L}_i\left({\beta}_0,\sigma \right)=\int \prod \limits_{j=1}^{N_i}P{\left({Y}_{ij}=1|{u}_i\right)}^{y_{ij}}{\left(1-P\left({Y}_{ij}=1|{u}_i\right)\right)}^{\left(1-{y}_{ij}\right)}f\left({u}_i;{\sigma}^2\right)d{u}_i$, where *N* is the total number of families, *N*_*i*_ is the number of family members of family *i* and *f* is the density function of *u*_*i*_. Maximization of the likelihood cannot be analytically solved and requires numerical approximation techniques (e.g. quadrature methods).

Finally, we can obtain ${\hat{p}}_i$, the expected value of *p*_*i*_ given the observed data of family *i* and the estimated *β*_0_ and *σ*, denoted by ${\hat{\beta}}_o$ and $\hat{\sigma}$, as

3 $$ {\hat{p}}_i={\int}_{-\infty}^{\infty}\frac{e^{{\hat{\beta}}_0+u}}{1+{e}^{{\hat{\beta}}_0+u}}\ f\left(u|{y}_{i1},\dots, {y}_{i{N}_i},{\hat{\beta}}_0,\hat{\sigma}\right) du $$

where $f\left(u|{y}_{i1},\dots, {y}_{i{N}_i},{\hat{\beta}}_0,\hat{\sigma}\right)$ is the density of the posterior distribution of the family specific random effect. Using Bayes’ rule, this density can be obtained as

$$ f\left(u|{y}_{i1},\dots, {y}_{i{N}_i},{\hat{\beta}}_0,\hat{\sigma}\right)=\frac{f\left({y}_{i1},\dots, {y}_{i{N}_i}|{\hat{\beta}}_0,u\right)f\left(u|\hat{\sigma}\right)}{\int_{-\infty}^{\infty }f\left({y}_{i1},\dots, {y}_{i{N}_i}|{\hat{\beta}}_0,u\right)f\left(u|\hat{\sigma}\right) du} $$

where $$ f\left({y}_{i1},\dots, {y}_{i{N}_i}|{\hat{\beta}}_0,u\right)=\prod \limits_{j=1}^{N_i}P{\left({Y}_{ij}=1|{u}_i\right)}^{y_{ij}}{\left(1-P\left({Y}_{ij}=1|{u}_i\right)\right)}^{\left(1-{y}_{ij}\right)} $$.

We propose to consider ${\hat{p}}_i$ as a new longevity family score of family *i*, and we denote it by *mLRC*_*i*_. In this way, *mLRC* can be regarded as a model-based version of *LRC* which includes shrinkage based on *N*_*i*_. *mLRC*_*i*_ can still be interpreted as the proportion of long-lived members of family *i* but it captures the uncertainty due to family size by the different ‘weight’ each family receives through its estimated random effect ${\hat{u}}_i$.

### Software implementation

The new *mLRC* family score, together with the *LRC* and *FLoSS* have been implemented in R. The code is provided as supplementary material.

## Results

### Simulation study

Simulated data is generated under the assumption that a latent factor, shared by the members of the same family, controls the degree of longevity of the family. Based on the simulated data, we can measure the level of agreement between the underlying longevity factor and different longevity family scores.

Characteristics of the simulated datasets such as the number and size of the families are chosen to mimic our real data set. In each run of the simulation, we simulated *N* = 1000 families of different sizes, namely 200 families with respectively size 2,3,8,10, and 14 individuals. For each individual *j* of family *i*, where *i* = 1*,...,N,* we sampled survival percentiles *p*_*ij*_ from a beta distribution with parameters *a* = exp.(0*.*1) and *b* = *a* × exp.(−(1 + *u*_*i*_)), where *u*_*i*_ was a random effect common to the *N*_*i*_ members of family *i*. The random effect was sampled from a normal distribution with mean 0 and standard deviation 2. Large values of *u*_*i*_ decreased the survival percentile *p*_*ij*_, which meant that the families with the lowest values of the random effect were the most enriched for longevity.

For each family, we computed the *LRC* score and the new model-based *LRC* (*mLRC*). Both scores were compared in terms of their relation with family size and performance as selection tools. The simulation was repeated 1000 times.

**Fig. 2:**
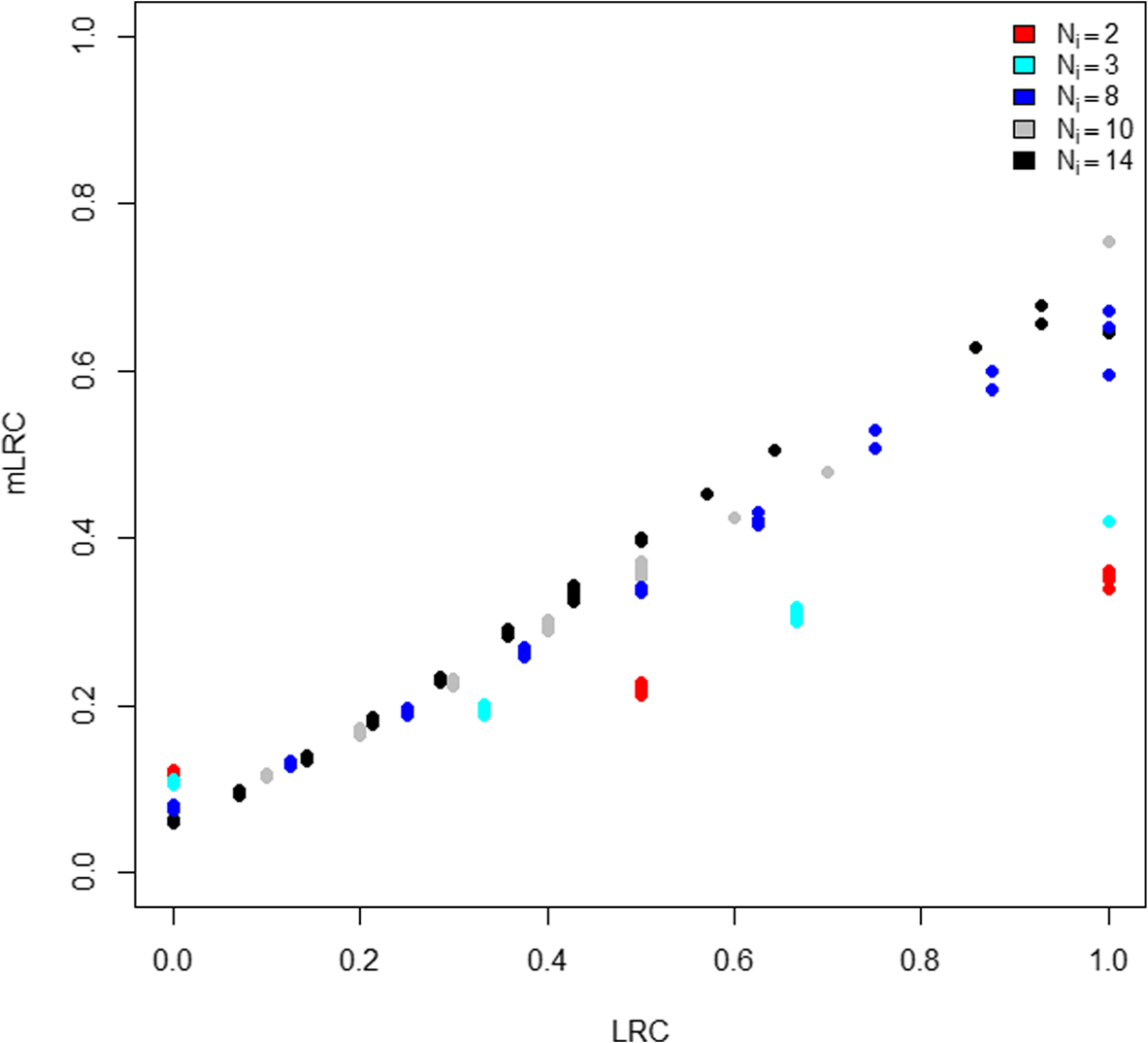
Comparison of *LRC* and *mLRC* with simulated data. For each of the *N* = 1000 families in one simulation run, we display the *LRC* score (x-axis) against the *mLRC* score (y-axis). Every point in the graphic represents a family, colored according to its size. Red dots represent families of size N_i_ = 2, light blue dots represent families of size N_i_ = 3, dark blue dots represent families of size N_i_ = 8, grey dots represent families of size N_i_ = 10 and black dots represent families of size N_i_ = 14

Table 1 shows the distribution of family size according to the values of *LRC* and *mLRC*. The *LRC* score is strongly affected by family size; families with low sibship sizes tend to have large values of *LRC* (left column of Table 1). No clear relation between family size and *mLRC* is observed (right column of Table 1), which is in agreement with the data generation mechanism. Figure 2 shows the comparison between the *LRC* and *mLRC* for all the families in one simulation run. For small families, the *mLRC* score is typically lower than the *LRC* score when the *LRC* score is large. This is caused by the penalization of our new method due to lack of information in small families. Analogously, small families are weighted upwards when the *LRC* score is low following the same principle of major uncertainty when the family size is small. Still, if the level of exceptionality of the observed family members is large, small families can still outperform large families. This is illustrated by small families (for example, with *N*_*i*_ = 2, red dots) appearing at the right part of the graphic in Fig. 2. The ability of *mLRC* to correctly deal with differences in family size, explains that the association between family size and the *mLRC* score is very low (right column Table 1).

**Table 1:** Family size and family scores in simulated data

Category	*LRC*	*mLRC*
[0,0.1]	10 (10–14)	10 (8–10)
(0.1,0.2]	10 (8–10)	3 (3–8)
(0.2,0.3]	10 (8–14)	3 (2–10)
(0.3,0.4]	3 (3–3)	5.5 (2–10)
(0.4,0.5]	2 (2–2)	8 (2–14)
(0.5,1]	3 (3–10)	10 (8–14)

In each of the 1000 simulation runs, LRC and mLRC were categorized in 6 groups (using 0.1,0.2,0.3,0.4 and 0.5 as cut-offs) and median family size in each group was calculated. As a summary over the 1000 simulation runs, we provide median and range (in brackets) of these values. The left column reports results based on LRC and the right column reports results based on mLRC

To evaluate the performance of selection rules based on the *LRC* and *mLRC* scores, we considered two definitions of longevity. First, the 10% of families with the lowest value of the random effect *u* were defined as truly long-lived. Second, we considered the 5% of families with the lowest value of the random effect *u* as truly long-lived*.* For both definitions, we checked the agreement between the truly long-lived families and the selected families based on the *LRC* and *mLRC* scores. To perform this selection, the families with the 10% (respectively 5%) largest *LRC* or *mLRC* score were labeled as long-lived. Since our main interest was to avoid families not enriched for longevity in our selection, we used the positive predictive value (PPV) as summary measure of our simulations. The PPV is defined as the proportion of truly long-lived families among those classified as long-lived using the score-based selection rule under investigation.

**Fig. 3:**
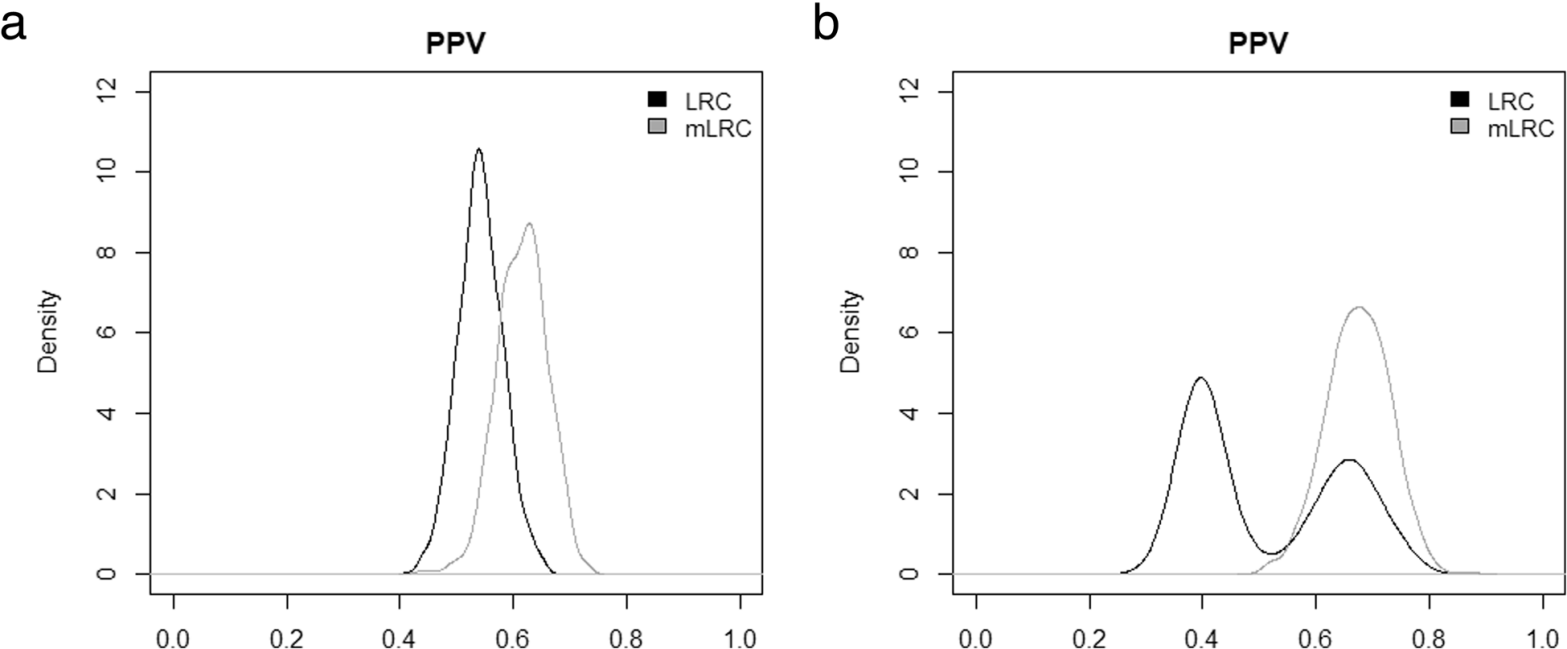
Evaluation of *LRC* and *mLRC* as selection tools with simulated data. Distribution of positive predictive (PPV) values across 1000 simulation runs. For each simulation run, the PPV associated to the selection rule under investigation was computed. Black lines represent the results based on *LRC* and grey lines represent the results based on *mLRC*. The left panel shows the results when defining the 10% of families with the lowest value of the random effect u as truly long-lived and the selection criterion is declaring families with the 10% largest values of the score as long-lived. The right panel shows the results for the more strict definition of longevity, based on the 5% lowest values of the random effect u and the selection criterion is declaring families with the 5% largest values of the score as long-lived

Figure 3 shows the distribution of the positive predictive values from the 1000 simulation runs. When defining the 10% of families with the lowest value of the random effect *u* as truly long-lived (left panel of Fig. 3), the mean PPV for the selection based on *LRC* was 54% (sd = 4%), meaning that on average, among the 1000 top 10% families classified as long-lived according to *LRC*, 54% were truly long-lived. The mean PPV increased to 62% (sd = 4%) when using *mLRC* for selection of the top 10% families. If we focus on the top 5% families (right panel of Fig. 3), the average accuracy of the selection based on *LRC* decreased (mean PPV = 0.52,sd = 0.13). In addition, we found large variability of the PPV among simulation runs, which indicates instable performance of the *LRC* score. On the contrary, the accuracy based on *mLRC* increased in this case (mean PPV = 0.67, sd = 0.06). These results show that selection of families based on *mLRC* clearly outperforms selection based on *LRC*.

### Real data: the historical sample of the Netherlands

The Historical Sample of the Netherlands (HSN) Long Lives study [27, 28] is an extensive database which contains lifetime data for the members of 1326 five-generational families, evolving around a single proband (Index Person, IP) per family [29]. We focus on the siblings present in the second (F2) generation which are the children of the IPs. The selection for a part of these IPs was enriched for longevity. Specifically, the selected IPs were part of a case-control study to compare differences in longevity among descendants of 884 IPs who died at 80 years or beyond (case group) and 442 IPs who died between 40 and 59 years (control group) [18, 30]. After removing individuals with missing age at death, single child sibships, and individuals belonging to non-extinct birth cohorts by the date of data collection (death dates were updated at 2017 and 110 years was set as maximum age); the final sample of our analysis consisted of 1105 sibships, children of the aforementioned HSN IPs, which corresponded to 5361 individuals.

To evaluate the performance of the new longevity family score *mLRC* and compare it to the original *LRC*, we first randomly selected a sample of independent individuals by choosing one individual at random from each of the 1105 available sibships. This set of independent individuals was set aside from the score calculations and subsequently used as a validation set to evaluate score performance. This validation set resembles the potential candidates to be included in, for example, a GWA study of longevity. Then, *LRC* and *mLRC* were calculated based on a sample of 4256 individuals. Afterwards, based on both scores we conducted a selection of long-lived families and we checked if those corresponded with a survival benefit in the validation set using Cox proportional hazard regression.

**Fig. 4:**
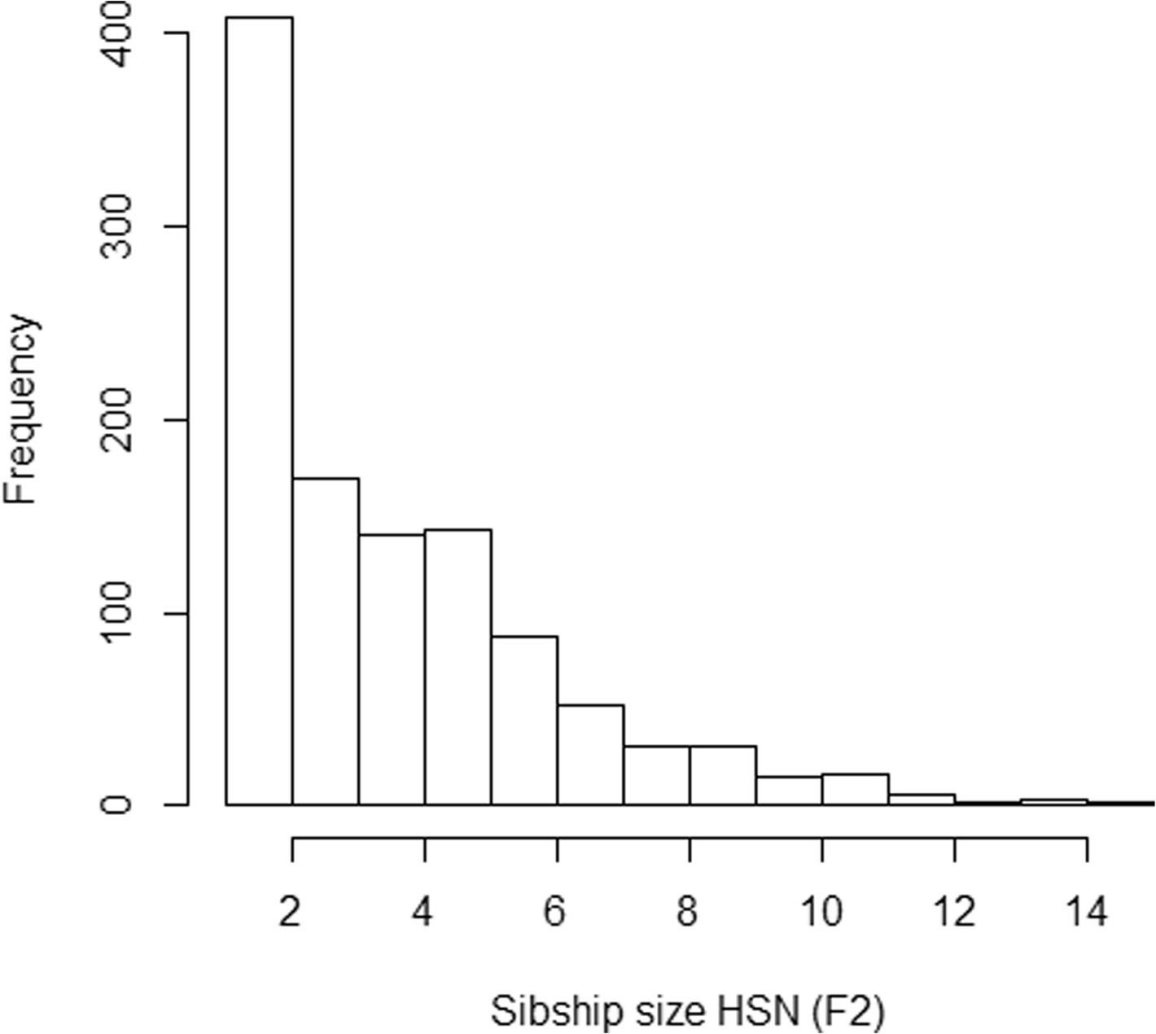
Sibship size in the HSN data

**Fig. 5:**
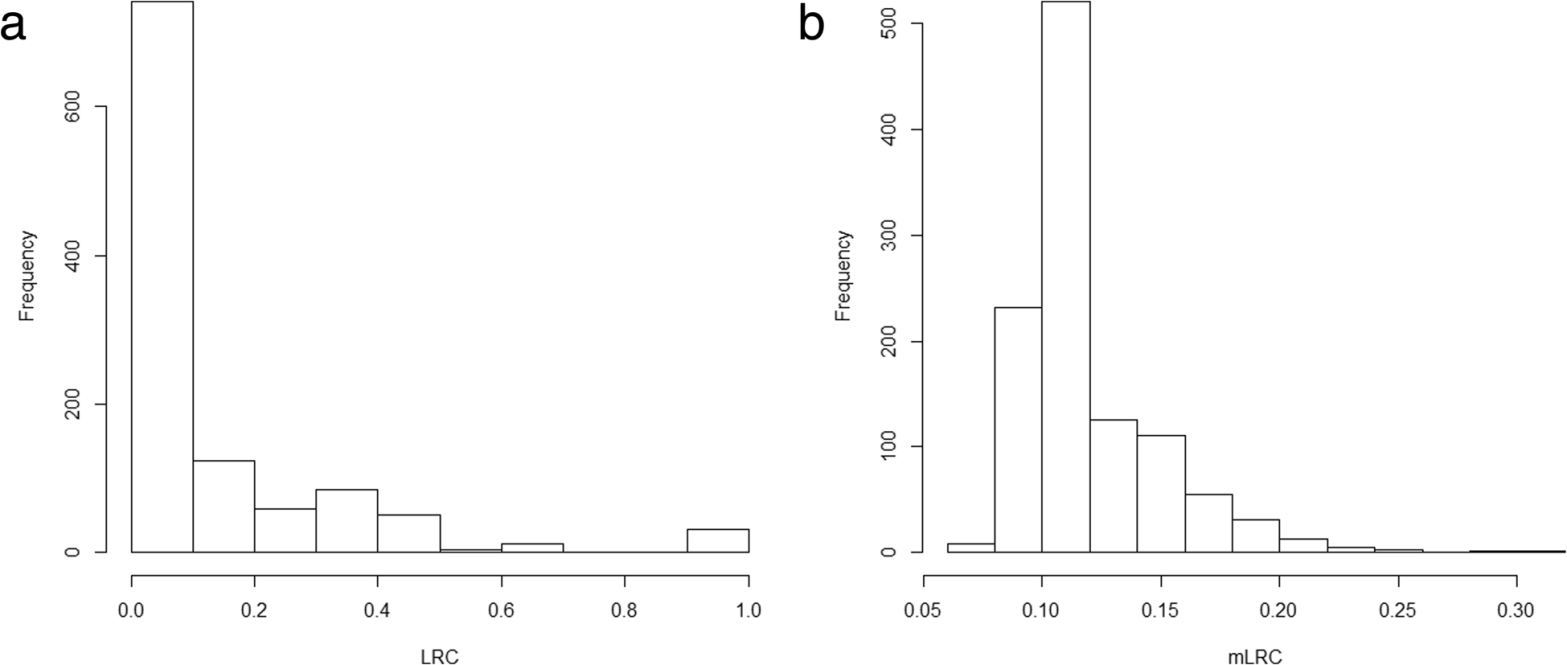
Distribution of the *LRC* (left panel) and *mLRC* (right panel) scores in the analyzed sibships of the HSN dataset

The sibship size was highly varying in the sample (Fig. 4). As expected, *LRC* is largely affected by family size, and families with large values of *LRC* present lower sibship sizes (Table 2). We do not observe a pattern in family size according to the estimated level of familiar longevity using *mLRC*. Figure 5 shows the distribution of the *LRC* and *mLRC* scores in the analyzed sibships of the HSN dataset.

**Table 2:** Family size and family scores in the HSN data

Category	*LRC*	*mLRC*
[0,0.1]	11	5
(0.1,0.2]	6	3
(0.2,0.3]	4	4
(0.3,0.4]	3	6
(0.4,0.5]	2	–
(0.5,1]	1	–

Median family size according to longevity family scores values of LRC and mLRC. Each scores were categorized in 6 groups (using 0.1,0.2,0.3,0.4 and 0.5 as cut-offs) and median sibship sizes are reported for each group. The left column reports results based on LRC and the right column reports results based on mLRC

Previous literature [18], has suggested *LRC* ≥ 0.3 as a selection criterion to capture the heritable longevity trait. In our sample, *LRC* ≥ 0.3 corresponds to the selection of the 15% families with the largest values of the *LRC* score. We evaluated the performance of this selection criterion by comparing the survival of the individuals of the validation set belonging the selected families to the rest of individuals in the validation set. Analogously, we selected the top 15% families according to ranking resulting from using the *mLRC* as longevity score which corresponds to define families with *mLRC* ≥ 0.15 as long-lived and evaluated this selection strategy using the validation set. For each of the proposed selections, we fitted a Cox regression model with the each of the selection indicators as explanatory variables. Both models were adjusted by gender and birth cohort. Table 3 shows that the selection of long-lived individuals based on the *mLRC* score predicts excess survival in the validation set better than the selection based on the LRC score (*β*_*LRC ≥ 0.3*_ = − 0*.287*, *β*_*mLRC ≥ 0.15*_ = − 0*.*321).

**Table 3:** Evaluation of selection strategies of long-lived families based on LRC and mLRC scores in the HSN

*Score*	*β*	s.e.
*LRC ≥ 0.3*	−0.287	0.082
*mLRC ≥ 0.15*	−0.321	0.084

Long-lived families were defined as those belonging to the top 15% of each score which corresponded to a cut-off of 0.3 in LRC and a cut-off of 0.15 in mLRC. For each binary variable defined in these cut-offs, a multivariable Cox proportional hazard regression model corrected by birth cohort and gender is fitted in the validation set. Estimates of the resulting regression coefficient(β) and standard error (s.e.) are reported

## Discussion

We proposed a method based on mixed effects regression modelling to estimate longevity family scores and properly account for differences in family size when ranking families according to their longevity and use this ranking for the selection of participants in longevity studies. Our simulation study and real data analysis show that the new proposed approach (*mLRC*) yields better results than its empirical counterpart (*LRC*) in terms of selection of long-lived individuals. We showed that the *SE*_*f*_ score and *FLoSS* increase with the addition of non-long-lived family members and their interpretation is ruled by the underlying family size distribution. We also showed that the *LRC* score puts too much weight on small, less-informative families. The *mLRC* score was not affected by sibship size and therefore its resulting ranking better predicted the survival of 1105 independent study participants. The new *mLRC* score seems to reduce heterogeneity in the selection of families and its application could potentially help to improve power and bias reduction in longevity studies.

Our current approach has some limitations. First, the binary nature of the current *mLRC* discards important information which could contribute to improve its performance. An interesting property of the *SE*_*f*_ score and the *FLoSS* is their continuous nature. Other continuous longevity family scores have been previously proposed [4, 24, 25]. The Longevity Family Score (*LFS*) [4] and the Family Mortality History Score (*FMHS*) [25] are closely related to the *SE*_*f*_ and *FLoSS* since all use the same measure of individual survival exceptionality based on transforming the observed ages at death to survival percentiles in a reference population using life tables. The *FMHS* is restricted to parental data and hence not subject to differential family size. The *LFS*, the *SE*_*f*_ and the *FLoSS* are extensions of the *FMHS* which can deal with sibships of arbitrary size. The Familial Excess Longevity (*FEL*) score [24] is also continuous but it does rely on population life tables. Instead, individual survival exceptionality is defined as the difference between observed and expected age, derived from an accelerated failure time regression model. Both the *LFS* and the *FEL* scores are based on the mean as family-specific summary measure and hence share with the LRC score the discussed limitations of empirical expectations.

A potential drawback of all these continuous longevity scores is that relatively young family members can contribute positively to these scores. Even after conditioning on being older than 40 as proposed for the *FLoSS*, the resulting score is probably influenced by ages at death which are not extreme enough to capture the heritable longevity trait. Evidence of this is supported by studies that have pointed towards increasing family aggregation of survival when focusing on more extreme ages at death for longevity definition [13, 31] and recent publications indicating that the longevity trait seems to be heritable considering lifespan thresholds beyond the top 10% survivors of a given birth cohort [5]. A model-based modified version of *SE*_*f*_ or the *LFS* which minimizes the contribution of young family members seems a promising line of future research. However, the extremely skewed distribution of the individual measure of longevity of these scores makes the extension of our method not straightforward.

Another important topic is dealing with alive or lost on follow-up (right censored) individuals when constructing longevity family scores. We have assumed full observation of lifespan of siblings included in the calculation of the score, so scores can be regarded as family history scores of alive relatives who could potentially be selected to participate in a (genetic) longevity study.

The *FLoSS* score is the extension of the discussed score *SE*_*f*_ to allow for the inclusion of right censored observations. The *FLoSS* follows a single imputation approach based on imputing alive individuals with the sex and birth cohort specific conditional expected age at death. This is an example of single imputation which underestimates the uncertainty of estimates and can potentially lead to bias. More advanced methods are possible in the mixed effect setting and its inclusion is left as subject of future research. Finally, recent evidence [9] indicates that the inclusion of family members of different degree of relatedness is of great importance to capture the heritable longevity phenotype and hence the proposed method should also be extended to this more complex setting.

Finally, it is important to mention that our approach may result in selections that are influenced by family-shared non-genetic factors. Despite previous research based on historical pedigree data have led to little evidence for associations between non-genetic factors such as socio-economic status, fertility factors or religious denomination and familial longevity [5, 8–10], other socio-behavioral and environmental factors such as personality and lifestyle may influence familial clustering of longevity. Since many of these also have a strong genetic component itself it is most likely that gene environmental interactions can explain a part of the familial clustering of longevity. Still in this complex setting, the use of well-designed family scores is expected to reduce genetic heterogeneity and contribute to a power increase in case-control longevity studies to detect novel genetic loci. Moreover, our mLRC score can be applied in more general longevity studies devoted to investigate the interplay among genetic and non-genetic factors involved in longevity.

## Conclusions

To properly account for differences in family size is of paramount importance when deriving family scores of longevity and using them for ranking families and selecting participants in longevity studies. The methodology described in this paper is therefore of great relevance and can help to improve selection of participants in future longevity studies.

## Supplementary Information

**Additional file 1.**

## Data Availability

The data used for this study will be made freely available at the Data Archiving and Networked Services (DANS) repository but are currently not yet publicly available due to ongoing checks to guarantee that the data sharing process is in accordance with Dutch and international privacy legislation. Data are however available from the authors upon reasonable request.

## Abbreviations

FLoSS: Family Longevity Selection Score
FMHS: Family Mortality History Score
HSN: Historical Sample of the Netherlands
IP: Index person
LFS: Longevity Family Score
LRC: Longevity Relatives Count
mLRC: model-based Longevity Relatives Count
PPV: Positive predictive value
SE_f_: Survival Exceptionality
Sd: Standard deviation
